# LncRNAs: New Players in Apoptosis Control

**DOI:** 10.1155/2014/473857

**Published:** 2014-01-30

**Authors:** Marianna Nicoletta Rossi, Fabrizio Antonangeli

**Affiliations:** ^1^Pasteur Institute-Fondazione Cenci Bolognetti, Department of Cellular Biotechnologies and Hematology, Sapienza University of Rome, Viale Regina Elena 324, 00161 Rome, Italy; ^2^Pasteur Institute-Fondazione Cenci Bolognetti, Department of Molecular Medicine, Sapienza University of Rome, Viale Regina Elena 291, 00161 Rome, Italy

## Abstract

The discovery that the mammalian genome is largely transcribed and that almost half of the polyadenylated RNAs is composed of noncoding RNAs has attracted the attention of the scientific community. Growing amount of data suggests that long noncoding RNAs (lncRNAs) are a new class of regulators involved not only in physiological processes, such as imprinting and differentiation, but also in cancer progression and neurodegeneration. Apoptosis is a well regulated type of programmed cell death necessary for correct organ development and tissue homeostasis. Indeed, cancer cells often show an inhibition of the apoptotic pathways and it is now emerging that overexpression or downregulation of different lncRNAs in specific types of tumors sensitize cancer cells to apoptotic stimuli. In this review we summarize the latest studies on lncRNAs and apoptosis with major attention to those performed in cancer cells and in healthy cells upon differentiation. We discuss the new perspectives of using lncRNAs as targets of anticancer drugs. Finally, considering that lncRNA levels have been reported to have a correlation with specific cancer types, we argue the possibility of using lncRNAs as tumor biomarkers.

## 1. Introduction

Apoptosis is the most common type of programmed cell death by which the body eliminates damaged or exceeding cells without local inflammation. Thus, functional apoptotic pathways are essential for organ development and tissue homeostasis. DNA damage or growth factor's withdrawal can induce apoptosis through the socalled intrinsic pathway by the release of cytochrome c and other proteins from the intermembranous space of mitochondria [1]. Alternatively, the socalled extrinsic apoptotic pathway is triggered by the activation of specific death receptors on the cellular membrane [2]. Accordingly, deregulation of apoptosis is implicated in a wide range of diseases. Low rate of apoptosis can promote the survival and accumulation of abnormal cells, leading to cancer development or autoimmune disease [3, 4]. On the other hand, increased levels of apoptosis are associated with neurodegenerative diseases, characterized by progressive neuronal death, or with acute pathologies such as cardiac ischemia [5, 6]. During last years, much effort has been spent to study and possibly control apoptosis in pathological conditions. To this aim it is of fundamental importance to understand the molecular pathways and cellular stimuli that regulate and trigger apoptosis.

Genomic studies conducted in the past decades highlighted the presence of a large amount of DNA that is transcribed but not translated, leading to the formation of RNAs that do not code for proteins (noncoding RNAs) [7–9]. Some of these RNAs are associated with the translational machinery, such as ribosomal and transfer RNAs, but for many others a key role in the regulation of cell fate has been demonstrated [10, 11]. This class of regulatory RNAs includes not only the well-known microRNAs (miRNAs) but also an heterogenous group of socalled long noncoding RNAs (lncRNAs).

LncRNAs can be very different in size, ranging from 340 nucleotides of 7SK to 118 kb of Airn. They can be transcribed by RNA polymerase II or III, they can be either spliced or not, and localized either in the nucleus or in the cytoplasm. On the basis of the position of their genes they can be divided in long intergenic noncoding RNAs (lincRNAs) and in antisense RNAs (asRNAs) if they are transcribed from the minus strand of an open reading frame (for a review on lncRNA classification see [12]).

Regarding their functions, many nuclear lncRNAs are directly involved in gene expression control and several mechanisms of action have been demonstrated so far. Some lncRNAs act as downregulators of gene expression recruiting gene silencing complexes such as PRC1 and PRC2 to the promoters of target genes. This mechanism of action has been described for the well-known regulator of imprinting Xist that remains tethered to its site of transcription [13] and for HOTAIR that instead acts in *trans* [14]. Other lncRNAs, such as GAS5, act as decoy precluding the access of regulatory proteins to DNA [15]. Some lncRNAs modify the activity of DNA binding proteins changing the expression of target genes (e.g., CCND1) [16]. In the cytoplasm, lncRNAs have been described to modulate mRNA stability, for example, by duplexing with the 3′ UTRs [17], or to act as miRNA decoy, as it has been demonstrated for lincMD1 that sponges miRNA-133 and miRNA-135 during muscle differentiation [18].

Through those different mechanisms of action, lncRNAs are involved in the regulation of different aspects of both cell physiology and pathology, such as imprinting [19], maintenance of pluripotency [20], and cancer [21]. Indeed, the emerging view from recent studies and transcriptome analysis is that lncRNAs are often deregulated in cancer cells compared to normal cells, thus suggesting to exploit lncRNAs as potential cancer markers. Furthermore, their modulation (overexpression or downregulation according to the specific lncRNA) in cancer cells often induces apoptosis or sensitizes cells to apoptotic treatments, suggesting that lncRNAs can be considered, at least for some cancer types, as therapeutic targets. This review focuses on the role of lncRNAs in the apoptosis processes with particular attention to the studies performed on cancer cell lines and tissues.

## 2. Regulation of the Tumor Suppressor Genes *PTEN* and *p53* by lncRNAs

**Figure 1:**
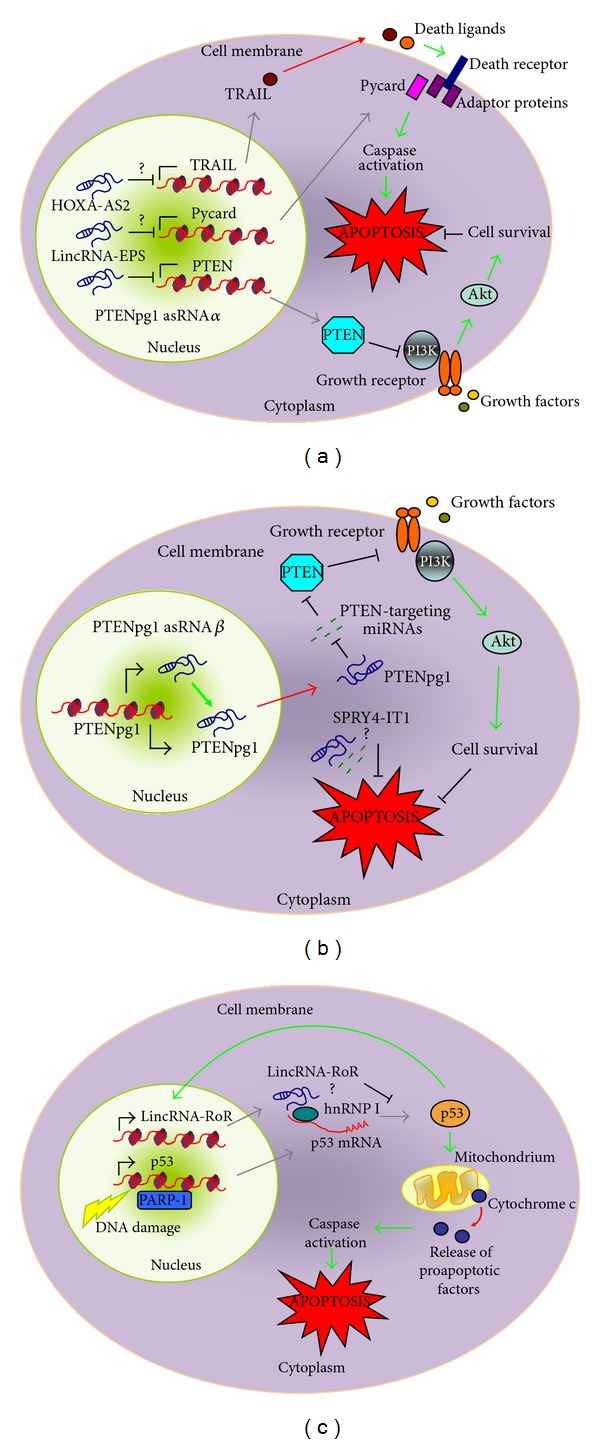
Schematic view of the different mechanisms through which lncRNAs can modulate apoptosis. (a) Transcriptional inhibition, as reported for PTENpg1 asRNA and proposed for HOXA-AS2 and LincRNA-EPS towards *PTEN*, *TRAIL,* and *Pycard* genes, respectively. (b) miRNA sponge. PTENpg1 can function as decoy for PTEN mRNA-targeting miRNAs; similar mechanism has been supposed for SPRY4-IT1. (c) Inhibition of mRNA translation. LincRNA-RoR has been hypothesized to interact with the mRNA binding protein hnRNP1 modulating the translation of p53 mRNA. L-bar arrows indicate gene transcription; T-bar arrows indicate negative regulation; green arrows indicate positive regulation; red arrows indicate release/translocation; grey arrows indicate mRNA translocation/translation; and ? indicates a supposed mechanism.

*PTEN* and *p53* are two of the most studied tumor suppressor genes and both of them have well described antiproliferative and proapoptotic activity [22, 23]. PTEN is able to induce apoptosis through the AKT/PI3 K pathway [24] and is epigenetically silenced in several cancers [25]. In 2010, Poliseno and colleagues demonstrated that PTEN mRNA is regulated by PTEN pseudogene1 (PTENpg1), a lncRNA that sequesters numerous PTEN-targeting miRNAs by acting as miRNA sponge [26]. Moreover, Johnsson and colleagues demonstrated the presence of two antisense transcripts from the *PTENpg1 *gene, adding another level of complexity to the transcriptional and posttranscriptional regulation of PTEN [27]. In detail, one antisense transcript, the isoform *α* (PTENpg1 asRNA*α*) directly binds PTEN promoter and represses the transcription of the gene (Figure 1(a)), while the isoform *β* stabilizes and facilitates the export in the cytoplasm of PTENpg1, enhancing its role of miRNA sponge and thus increasing PTEN mRNA stability and translation (Figure 1(b)). Accordingly, suppression of PTENpg1 asRNA*α* sensitizes cells to the DNA damaging agent doxorubicin. This mechanism results in both transcriptional and posttranscriptional regulation of PTEN by its pseudogene.

p53 is a well-known tumor suppressor that regulates many cellular processes including DNA repair, cell cycle progression, and apoptosis [22]. Consistently with its importance, p53 expression is subjected to different levels of regulation: transcriptional, posttranscriptional, and translational. It was recently demonstrated another level of regulation for p53 by lincRNA-RoR (RoR) [28]. In particular, after DNA damage stimuli, ectopic overexpression of RoR was shown to downregulate p53 accumulation leading to decreased levels of apoptosis, as evaluated by TUNEL assay. The authors claim that RoR acts on newly synthesized p53 interacting with the RNA binding protein hnRNP I (Figure 1(c)). In addition, the authors also demonstrated a regulative feedback loop as p53 induces the transcription of RoR (Figure 1(c)).

## 3. LncRNAs Involved in Apoptosis of Cancer Cells

Several recent studies described an altered expression pattern of specific lncRNAs in cancer cells when compared with normal cells and tissues. Some of them reported lncRNAs being negative regulators of apoptosis in different types of tumors (Table 1). For example, AFAP1-AS1, a lncRNA derived from the antisense strand of the *AFAP1* coding gene locus, was shown to be hypomethylated and upregulated in esophageal adenocarcinoma tissues and cell lines [29]. Its silencing by small interfering RNA (siRNA) was reported to induce apoptosis in the esophageal adenocarcinoma OE-33 cell line, considering both annexin V flow cytometry assay and caspase-3 cleavage by Western blot. Cell cycle analysis was also performed after siRNA treatment revealing that knockdown of AFAP1-AS1 induces G2/M-phase arrest. Taken together these findings suggest that AFAP1-AS1 can modulate both proliferation and programmed cell death in esophageal cancer cells.

Similar findings were reported by Khaitan and colleagues in melanoma cells [30]. Melanoma is the most common skin cancer and the authors reported the upregulation of the lncRNA SPRY4-IT1 in melanoma cells in comparison to melanocytes and keratinocytes. SPRY4-IT1 is transcribed from the second intron of the *SPRY4* gene and the two transcripts share similar expression profile, suggesting they may either be transcribed independently from the same promoter or, alternatively, they may be transcribed as a single transcript with SPRY4-IT1 then being processed from the intron of SPRY4. The effects of SPRY4-IT1 knockdown on cell death was investigated in the melanoma cell line WM1552C. The authors showed an increase of annexin V positive cells, while no differences were observed in propidium iodide-positive cells, indicating that the knockdown of SPRY4-IT1 induces cell death primarily through apoptosis and not necrosis. Interestingly, the main subcellular localization of SPRY4-IT1 was reported to be cytoplasmic and on the basis of its localization the authors speculate that SPRY4-IT1 could function as a sponge for proteins or RNAs as reported for other cytoplasmic lncRNAs (see Section 1) (Figure 1(b)).

In prostate cancer cell lines and tissues, Cui and colleagues reported the overexpression of the lncRNA PlncRNA-1 [31]. Upregulated genes in cancer cells often play a role in tumor survival and progression and accordingly the knockdown of PlncRNA-1 by siRNA was shown to induce apoptosis in LNCaP cells, as observed by increased cleavage of PARP-1, a key component of the DNA damage response.

Notably, Zhao and colleagues demonstrated a mechanism through which the lncRNA HOXA-AS2 inhibits apoptosis in a model of promyelocytic leukemia [32]. *HOXA-AS2* gene is located between *HOXA3* and *HOXA4* genes on the antisense strand. Its transcript is expressed in human peripheral blood neutrophils and in NB4 cells, a human promyelocytic leukemia cell line derived from a patient with acute promyelocytic leukemia. NB4 cells treated with all *trans* retinoic acid (ATRA) are prone to undergo apoptosis through caspase activation. Interestingly, the authors found that ATRA-treated NB4 cells increased HOXA-AS2 expression. Performing HOXA-AS2 knockdown by short hairpin RNA (shRNA) they showed an increase in ATRA-induced apoptosis measured by annexin V binding and by activity and cleavage of caspase-3, caspase-8 and caspase-9. The involvement of the intrinsic apoptotic pathway was confirmed by the increased levels of BAX, the well-known proapoptotic protein of the BCL2 family. More remarkably, they discovered an increase in TRAIL (TNF-related apoptosis-inducing ligand) protein and mRNA expression after ATRA treatment in HOXA-AS2 knockdown cells. It is known that ATRA-induced cell death in NB4 cells is linked to paracrine production of TRAIL [35]. This suggests the involvement also of the extrinsic apoptotic pathway. Indeed caspase-8 activation is due, at least in part, to the paracrine effects of TRAIL, as TRAIL-neutralizing antibody partially blocks caspase-8 cleavage. These results indicate that the lncRNA HOXA-AS2 negatively regulates ATRA-induced TRAIL production and the authors suggest that HOXA-AS2 may directly affect the transcription of the TRAIL gene (Figure 1(a)).

Other authors have reported examples of lncRNAs with proapoptotic effects in different cancer cells (Table 1). Expression levels of the lncRNA uc002mbe.2 were found lower in human hepatocellular carcinoma (HCC) cells compared to normal human hepatocytes and adjacent noncancerous tissues [33]. The expression levels were rapidly restored, within hours, upon treatment with the histone deacetylase inhibitor trichostatin A (TSA) and positively correlated with the apoptotic effect of TSA on HCC cells. Accordingly, uc002mbe.2 knockdown by siRNA reduced TSA-induced apoptosis as revealed by TUNEL assay [33]. Thus, at least in HCC cells, uc002mbe.2 is involved in the TSA-induced apoptosis.

Finally, in prostate cancer cell lines, Pickard and colleagues reported a lncRNA transcribed from the growth arrest-specific 5 (*GAS5*) gene locus able to mediate apoptosis in UV-C irradiated 22RV1 or PC-3 cells [34]. Again, cell death, evaluated by TUNEL assay, was increased in cells transfected with GAS5 constructs and attenuated following downmodulation of GAS5 expression.

## 4. LncRNAs Involved in Apoptosis during Development and Differentiation

Beside the cited paper that have investigated the role of lncRNAs in modulating apoptosis in cancer cells, there are also a couple of papers describing lncRNAs as apoptosis regulator during cellular differentiation and organ development. Hu and colleagues reported lncRNA-mediated antiapoptotic activity in murine erythroid terminal differentiation [36]. Using murine foetal liver cells as model of erythropoiesis, the authors characterized an erythroid specific lincRNA called LincRNA-EPS (for LincRNA erythroid prosurvival). LincRNA-EPS was found to be highly induced in terminally differentiating erythroblasts considering the developmental markers CD71 and Ter119. Loss of function studies by shRNA revealed that inhibition of LincRNA-EPS induction resulted in apoptosis (evaluated by annexin V staining, caspase-3 activity and TUNEL assay) and arrest of proliferation of erythroid progenitors. Conversely, LincRNA-EPS ectopic expression by retroviral transduction was able to prevent apoptosis in erythroid cells starved for erythropoietin (Epo), an essential prosurvival and differentiating cytokine of the erythropoiesis system, even if it did not restore the terminal differentiation, as determined by the levels of haemoglobin. These findings and the timing of LincRNA-EPS induction are in agreement with an Epo-derived survival mechanism mediated by LincRNA-EPS. Remarkably, microarray analysis after LincRNA-EPS overexpression highlighted the repression of many proapoptotic genes, such as *Bad, Bax, Caspase-2* and -*6, Fadd, Pycard*, and with *Pycard* being the most affected. Pycard plays its role in apoptosis as adaptor protein for caspase activation and several evidences were shown to suggest Pycard as direct target of LincRNA-EPS. Pycard expression during normal erythropoiesis is inversely correlated with that of LincRNA-EPS. Pycard overexpression results in similar phenotypes on erythroid terminal differentiation as inhibition of LincRNA-EPS induction. Pycard knockdown mimics the antiapoptotic phenotype conferred by LincRNA-EPS ectopic expression in Epo-deprived erythroid cells. Finally, overexpression of Pycard suppresses the antiapoptotic phenotype mediated by ectopic expression of LincRNA-EPS. Considering the nuclear localization of LincRNA-EPS, Hu et al. conclude that the antiapoptotic ability of LincRNA-EPS is mediated through repressing the transcription of Pycard and perhaps through the epigenetic control of other genes involved in cell apoptosis (Figure 1(a)).

Finally, studying the fragile X syndrome, Khalil and colleagues identified a new 2.4 kb lncRNA that was named FMR4 [37]. Fragile X syndrome, the most common cause of inherited mental retardation, is caused by the expansion of CGG trinucleotide repeats in the 5′ UTR of the fragile X mental retardation 1 gene (*FMR1*) [38, 39]. The expansion of CGG repeats above 200 leads to the repression or silencing of *FMR1* and consequently to the absence of the fragile X mental retardation protein (FMRP). LncRNA FMR4, that is located upstream and likely shares a bidirectional promoter with *FMR1*, becomes silenced too as a result of the CGG expansion in the 5′ UTR of *FMR1* in fragile X syndrome. The authors analyzed the expression pattern of FMR4 in adult and foetal tissues finding FMR4 widely expressed, especially in the kidney and heart during embryonic development. The authors hypothesize that the foetal cardiac expression of FMR4 may be of functional relevance considering the fact that many patients with fragile X syndrome exhibit heart defects such as dilation of the aortic root and mitral valve prolapse [40]. Furthermore, they described that FMR4 regulate cell proliferation in HEK-293T cells. Knockdown of FMR4 resulted in cell cycle arrest and induction of apoptosis, while the overexpression of FMR4 led to an increase in cell proliferation, indicating that FMR4 has an antiapoptotic function in human cells.

## 5. LncRNAs as Therapeutic Targets and Biomarkers

As reported above, some lncRNAs and probably others that will be identified in the next years are involved in promoting or inhibiting apoptosis. This finding is of great relevance for the design of new drugs for the treatment of cancer and degenerative diseases. Targeting lncRNAs is challenging because it can potentially open a new field in drug development as they are completely different from proteins in both conformation and mechanism of action. Indeed, often they act as transcriptional repressors and so targeting a lncRNA can lead to the upregulation of tumour suppressors, growth factors, transcription factors and genes that are deficient in various genetic diseases, while the majority of currently available drugs exhibit an inhibitory mechanism of action. On the other hand, lncRNAs are particular appealing as drug targets because, once developed, the right technology for the delivery of their modulator can be applied with minor modifications to many lncRNAs. For example, H19 is a lncRNA with oncogenic properties, upregulated in a wide range of tumors. A plasmid carrying diphtheria toxin under the control of the H19 regulatory sequence has been developed to target cells overexpressing H19. Intratumoral injection of the plasmid was successfully applied in patients with bladder, ovarian, and pancreatic cancers to reduce tumor size [41]. Moreover, it was recently published an *in vivo* study in which targeting the asRNA BDNF-AS the authors obtained an increase in the levels of brain derived neural factor (BDNF) in mouse brain [42]. In this study they used single-stranded oligonucleotides named antago-NATs to target the asRNA. Antago-NATs can act by blocking the interactions of asRNA (or NAT: natural antisense transcript) with effector proteins and/or by causing RNAase H-mediated degradation of the antisense transcript. The authors used 16mer antago-NAT oligonucleotides with phosphorothioate-modified backbones and three locked nucleic acid (LNA) substitutions at each end to protect the molecules against exonuclease cleavage and increase affinity to the RNA target. Indeed, antago-NATs seem to be capable of inducing locus-specific upregulation as the expression of unrelated control genes, even neighbouring genes, seems to be unaffected.

Despite these examples, many problems still need to be solved for an extensive use of lncRNAs inhibitors in clinical studies such as off-target toxicity, delivery of oligonucleotides and lifetime administration [43].

Interestingly, lncRNAs often present an aberrant expression pattern in cancer cells, arising the question if they can be used as biomarker for diagnosis. The use of lncRNAs in diagnostics has intrinsic advantages over protein-coding RNAs because measurement of their expression directly represents the levels of the active molecule. In contrast, mRNA levels are only indirectly indicative of the levels of the functional product, the encoded protein. Furthermore, lncRNA levels may have a higher correlation with particular cancer types and thus be more useful as diagnostic tools. A recent analysis combined the expression profiles of more than 10 thousand lncRNAs with 1 thousand of tumors from 4 different cancer types with the aim to identify new biomarkers and potential drug targets. The authors identified and validated two new lncRNAs that drive prostate cancer progression [44]. LncRNAs are often stable in human serum and thus measuring either marker RNAs (e.g., by qPCR) or the entire transcriptome (e.g., RNA-seq) may allow the noninvasive generation of reliable and actionable clinical indicators [45]. For example, the lncRNA prostate cancer gene 3 (PCA3) is highly associated with prostate cancer and is routinely used to indicate prostate cancer risk from urine samples [46].

## 6. Conclusions

The world of lncRNAs has just started to be disclosed and only for a few of them the mechanisms of action are already known. Growing amount of evidences point out that lncRNAs are implicated in the control of apoptosis but their molecular roles in the apoptotic pathways are still largely unknown. Thus, it is now of primary importance not only to continue in identifying new lncRNAs involved in apoptosis but also to deepen the knowledge of mechanisms by which each lncRNA regulates apoptosis as well as other cellular processes. LncRNAs are now emerging as new master regulators of cell fate in response to stimuli and stress conditions and, as highlighted in this review, the outcome between cell proliferation and cell death is due, at least in certain types of cancer cells, to the expression levels of tissue-specific lncRNAs. Furthermore, lncRNA levels may have a correlation with particular cancer types and thus can be useful diagnostic tools as tumor biomarkers. The idea of targeting lncRNAs to modulate apoptosis in cancer cells is now opening a challenging field in drug discovery.

## Figures and Tables

**Table 1:** LncRNAs involved in the regulation of apoptosis in cancer cells.

LncRNA	Cancer cell line	Apoptotic effect	Reference
AFAP1-AS1	Esophageal adenocarcinoma OE-33	−	[29]
SPRY4-IT1	Melanoma WM1552C	−	[30]
PlncRNA-1	Prostate cancer LNCaP	−	[31]
HOXA-AS2	Promyelocytic leukemia NB4	−	[32]
uc002mbe.2	Hepatocellular carcinoma Huh7	+	[33]
GAS5	Prostate cancer PC-3	+	[34]

+: indicates proapoptotic effect; −: indicates antiapoptotic effect.
